# The increase in cancer prevalence and hospital burden in Western Australia, 1992–2011

**DOI:** 10.1186/s12963-014-0033-x

**Published:** 2014-12-19

**Authors:** Susannah Maxwell, Peter O’Leary, Terry Slevin, Rachael Moorin

**Affiliations:** Faculty of Health Sciences, Curtin University, 7 Parker Place, Bentley, Western Australia Australia; School of Pathology and Laboratory Medicine, The University of Western Australia, Crawley, Australia; School of Women’s and Infants’ Health, The University of Western Australia, Crawley, Australia; Clinical Biochemistry, PathWest Laboratory Medicine, Princess Margaret Hospital for Children, Western Australia, Australia; The Cancer Council Western Australia, 15 Bedbrook Place, Shenton Park, Western Australia 6008 Australia; Centre for Behavioural Research in Cancer Control, Curtin University, Perth, Western Australia Australia; Centre for Health Services Research, School of Population Health, University of Western Australia, 35 Stirling Highway Crawley, Western Australia, 6009 Australia

**Keywords:** Prevalence, Epidemiology, Cancer, Burden of disease, Health services

## Abstract

**Purpose:**

To describe cancer prevalence and hospital service utilization by prevalent cancer patients in Western Australia from 1992 to 2011.

**Methods:**

This study was a population-based cohort study using the Western Australia (WA) Cancer Registry (1982 to 2011) as the source of incident cancer cases. These data were linked to mortality (1982 to 2011) and hospital morbidity (1998 to 2011) records via the WA Data Linkage System to ascertain complete and limited-duration prevalence and cancer-related hospitalizations over time. Prevalence rates were calculated using estimated residential population data from the Australian Bureau of Statistics.

**Results:**

In 2011, one in every 27 people living in WA had been diagnosed with cancer at some time in their lifetime, and one in 68 had been diagnosed within the previous five years. Between 1992 and 2011, complete cancer prevalence in Western Australia increased by a magnitude of 2.5-fold. Forty-five and 44% of the increase in complete cancer prevalence in males and females between 1992 and 2011 can be attributed to prostate and breast cancer, respectively. The absolute number of cancer-related bed days increased 81 and 74% in males and females, respectively, diagnosed within one year, between 1998 and 2011.

**Conclusions:**

The prevalence of cancer and the burden it places on hospitals continues to rise, demanding ongoing efforts to prevent cancer through modifiable risk factors and better, more efficient use of health resources. Steps should to be taken to understand and address overdiagnosis and overtreatment.

## Introduction

Cancer is the leading cause of burden of disease in Australia as measured by mortality and disability [1] and the sixth-most expensive chronic disease, with cancer diagnosis, treatment, and care accounting for 7% of total health system expenditure [2]. While incidence and survival rates reported by cancer registries provide valuable information to assess the cancer burden and guide health system planning, they alone do not provide a complete picture of cancer in the community, and tend to focus on the burden of cancer within the first few years of diagnosis. As more people are living with and beyond cancer, cancer prevalence, the absolute number of people living in the community who have been diagnosed with cancer, [3,4] has become increasingly valuable for health service planning [4,5], just as it is for other chronic diseases such as diabetes. This is particularly so for long-term survivors (those who are at least five years beyond their diagnosis with a low chance of recurrence [6,7]), who have different health service needs due to the risk of late and long-term adverse effects of cancer treatment [6].

Prevalence can be “complete,” representing all individuals who have ever had a diagnosis of cancer, or of “limited duration,” describing the absolute number of individuals alive within a specified time period since diagnosis (such as for those diagnosed within five or 10 years). Limited duration prevalence can provide a convenient measure given the limited surveillance period of cancer registries, but it also recognizes the different stages of cancer management, with time since diagnosis being a reasonable indicator of phase of cancer care, from acute care following diagnosis through palliative care and death or to being a long-term survivor [8]. Prevalence can be estimated indirectly by modeling incidence, mortality, and survival data; however, more precise estimates can be gained with a direct method [9] using data on incident cases and death records to determine the number of individuals remaining alive at a certain point in time.

In Australia all cancer, with the exception of nonmelanoma skin cancer, is a notifiable disease. Cancer diagnoses are reported to the State Cancer Registry in Western Australia (WA), which provides annual population-based incidence and cancer-related mortality statistics [10]. Prevalence of cancer in WA has not been directly estimated since a 2002 study, which reported annual prevalence of cancer from 1990 to 1998 [4]. In this paper we directly estimate and describe trends in the size and composition of the cancer-prevalent population by cancer type and time since diagnosis between 1992 and 2011, as well as hospitalization trends of prevalent cases between 1998 and 2011. These data will allow health services to better allocate their resources and tailor activities to meet the needs of this population.

## Methods

This study was a population-based cohort study using the WA Cancer Registry (1982 to 2011) as the source of incident cancer cases. These data were linked to mortality (1982 to 2011) and hospital morbidity (1998 to 2011) records via the WA Data Linkage System [11] to ascertain person prevalence and cancer-related hospital service utilization in prevalent cases.

Incident cases were identified from 1 January 1982 to 31 December 2011 inclusive on the basis of tumour site code, morphology code, and behavior type. The study was restricted to people who were identified as residents of WA on their cancer registry record. Only invasive primary tumors were selected, with metastases from a previous primary tumour and benign and *in situ* neoplasms excluded. Where more than one record of the same cancer occurred in the same individual the earliest record was used as the incident case for that individual. Multiple records pertaining to different cancer types were not considered. Analyses were also conducted according to the type of cancer using the International Classification of Diseases for Oncology codes provided in the cancer registry data [12,13]. The types of cancer included were those commonly reported separately in the cancer incidence and mortality in Western Australia reports and are shown in Table 1.

**Table 1:** **Types of cancer analysed separately** [13]

**Cancer**	**ICD/ICD-O-3 Codes**
Bladder and urinary tract	C65-C68
Breast**	C50
Cervix**	C53
Colorectal	C18-C20, C218
Kidney	C64
Laryngeal	C32
Leukaemias (all)	9800-9801, 9805, 9836–9837, 9823, 9820, 9826, 9827, 9831–9834, 9840, 9861, 9866–9867, 9870–9874, 9891, 9895-9897, 9910, 9920, 9930-9931, 9863, 9875-9876, 9860, 9940, 9945-9946, 9948
Liver and intrahepatic bile ducts	C22
Lung, bronchus, trachea	C33
Lymphomas (all)	9590, 9650–9667, 9670–9671, 9673, 9675, 9678–9680, 9684, 9687, 9689–9691, 9695, 9698–9699,9766, 9700–9702, 9705, 9708–9709, 9714, 9716, 9717–9719, 9727–9729, 9591, 9596–9599,* 9687
Melanoma	C44; M-8720-8790
Mesothelioma	M905; ICD10 C45
Myeloma	9731-9734
Oesophageal	C15
Ovarian**	C56
Prostate*	C61
Stomach	C17
Testicular*	C62
Thyroid	C73
Uterine (corpus)**	C54

*male only **female only.

ICD-0–3 codes: *9597, *9598 and *9599 are Western Australian Cancer Registry codes for not otherwise specified non-Hodgkin Lymphoma which can be grouped as low, intermediate, or high grade, respectively, but which would only be otherwise placed in the ICD-O classification as code 9591.

The point prevalence of cancer was calculated at 30 June of each year (1992 to 2011) counting the number of incident cases diagnosed prior to this date who were not known to have died (using a combination of WA mortality records and the death indicator on the WA cancer record where available) as prevalent cases. Complete prevalence was calculated for males and females as well as limited duration prevalence [14] for those up to one year postdiagnosis, at one to five years, and at five to 10 years postdiagnosis for all cancers (Table 1). Rates were calculated using the number of persons resident in Western Australia at that time from the Australian Bureau of Statistics [15] as the denominator.

### Correction for underascertainment due to start of WA Cancer Registry

Since cancer diagnoses have only been recorded on the WA Cancer Registry since 1 January 1982, there was likely to be underascertainment of complete prevalence, especially in the early years. To estimate the number of cancer diagnoses underascertained it was assumed that a 29-year look-back (i.e., that the number of prevalent individuals in 2011) provided a true estimate (and thus the full count of the prevalence) of the number of individuals living with cancer.

The corrected number of prevalent individuals in the early years was estimated by (i) calculating the proportion of the true number of individuals that would have been estimated in 2011 using incrementally shorter (by one year) look-back periods and (ii) applying the appropriate proportion determined in part (i) to the prevalence calculated for look-back periods less than 29 years as shown in equation 1 below.

$$ \mathrm{C}\mathrm{P} = {\mathrm{P}}_{\mathrm{X}}/\left(1\hbox{--} \left(\left({\mathrm{P}}_{2011\_29\mathrm{yrs}}\hbox{--} {\mathrm{P}}_{2011\_\mathrm{Yyrs}}\right)/{\mathrm{P}}_{2011\_29\mathrm{yrs}}\right)\right) $$

Where CP = Corrected Prevalence, P = Prevalence, X = the year to be corrected, and Y = the number of years of look-back data available for year X.

The number of years of look-back (one to 29) and the calendar years (1982 to 2011) were then used to create a table of cumulatively adjusted correction factors for each year. These correction factors, together with the prevalence derived from the WA Cancer Registry data, were used to provide a corrected estimate of the prevalence of cancer for each year of the study. This correction was undertaken for all invasive cancers and for each type of cancer separately for each gender.

Data for complete prevalence and limited-duration prevalence for persons more than 10 years postdiagnosis are reported using these corrected estimates. Data presented for the remaining limited duration prevalence windows did not require correction.

### Hospitalizations

Linked hospital records were considered relevant to the treatment of cancer if they contained a principal diagnosis of the cancer under investigation or they contained procedure codes relating to chemotherapy or radiotherapy. The cumulative and average number of bed days and admissions per prevalent male and female up to one year postdiagnosis, at one to five years, and five to 10 years postdiagnosis were calculated using the number of prevalent individuals as the denominator.

This study was approved by the Western Australia Department of Health, Human Research Ethics Committee, which exempted the study from requiring individual patient consent.

## Results

### Cancer prevalence

**Figure 1:**
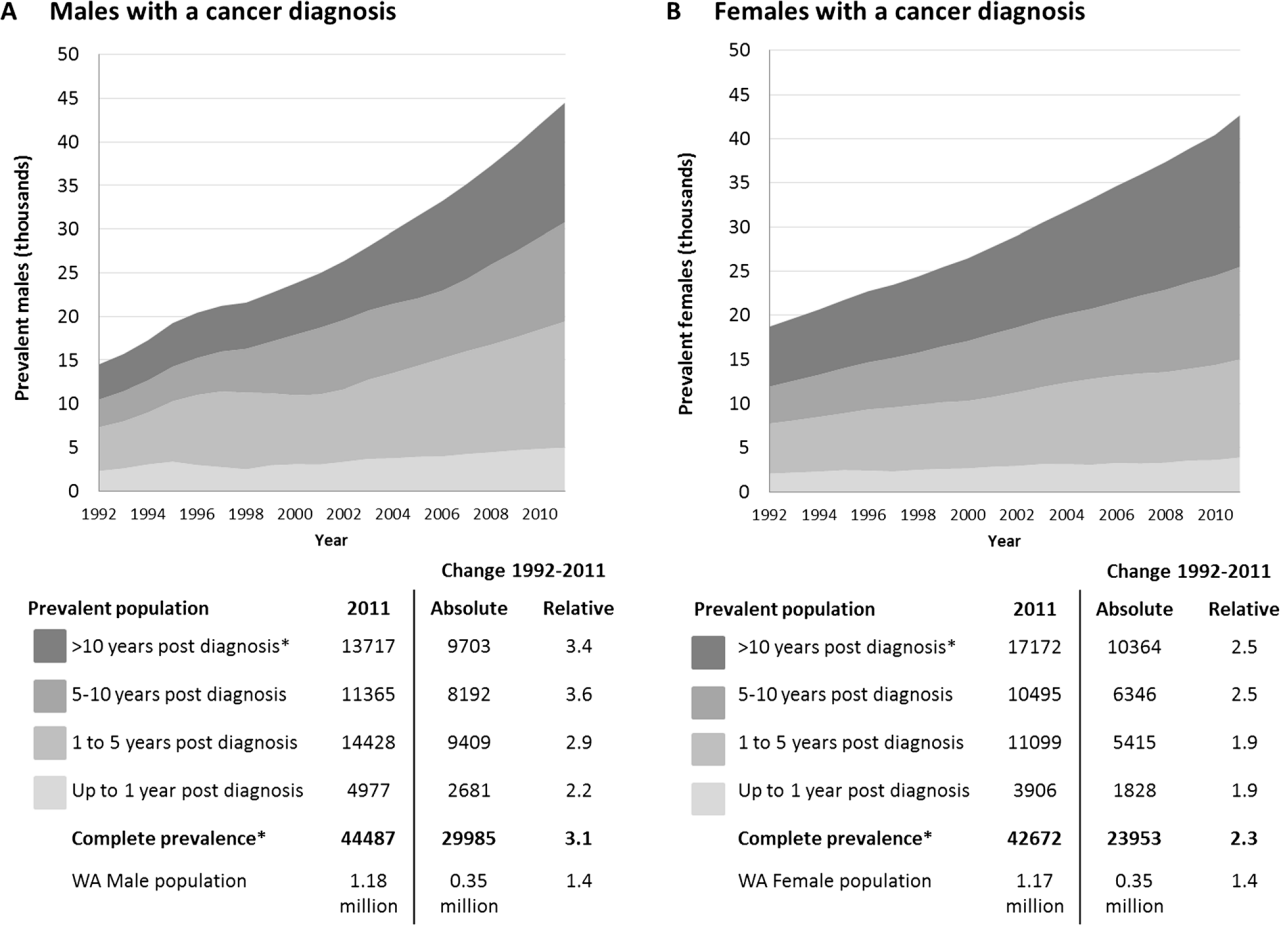
**Complete and limited-duration prevalence in males and females, 1992–2011.** Figure 1 shows the cumulative prevalence for all cancers for males **(A)** and females **(B)** between 1992 and 2011 by time since diagnosis; up to one year, one to five years, five to 10 years, and more than 10 years. The table immediately below the chart lists the absolute prevalence numbers for 2011, along with the absolute and relative change in cancer prevalence and the WA population between 1992 and 2011.

In 2011, 37.6 per 1,000 males (one in 27) and 36.5 per 1,000 females (one in 27) living in WA had a history of cancer, and 16.4 (one in 61) and 12.8 (one in 78) had been diagnosed within the previous five years (Table 2). Between 1992 and 2011, complete cancer prevalence increased by 29,985 males and by 23,953 females, representing a 2.5-fold increase in the estimated absolute number of surviving individuals who had ever been diagnosed with cancer in WA (Figure 1). During this time, the WA resident population increased by only 40% [15]. Over the same time period the absolute number of males and females alive more than 10 years postdiagnosis increased by 3.4- and 2.5-fold, respectively. The mix of cancer cases in the community by time since diagnosis has changed somewhat over time, with long-term survivors (more than five years since diagnosis) contributing 56% of all prevalent males and 65% of prevalent females in 2011, compared to 50% and 59% in 1992 (Table 2).

**Table 2:** **Complete, limited-duration, and specific cancer site prevalence per 1,000 males and females, 1992 and 2011**

			Per 1,000 (proportion**)		
			**1992**	**2011**	**Relative change**
**Males**	Complete prevalence*		17.4	37.6	2.2
	**Duration**	>10 years*	4.8 (28%)	11.6 (31%)	2.4
		**5–10 years**	3.8 (22%)	9.6 (26%)	2.5
		**1–5 years**	6.0 (35%)	12.2 (32%)	2.0
		**Up to 1 year**	2.7 (16%)	4.2 (11%)	1.5
	Cancer site* Prostate		3.0 (17%)	13.5 (36%)	4.5
		**Melanoma**	3.7 (21%)	7.0 (19%)	1.9
		**Colorectal**	2.6 (15%)	4.5 (12%)	1.7
**Females**	Complete prevalence*		22.71	36.5	1.6
	**Duration**	> 10 years*	8.3 (36%)	14.7 (40%)	1.8
		**5–10 years**	5.0 (22%)	9.0 (25%)	1.8
		**1–5 years**	6.9 (30%)	9.5 (26%)	1.4
		**Up to 1 year**	2.5 (11%)	3.3 (9%)	1.3
	Cancer site*	**Breast**	7.6 (34%)	14.4 (39%)	2.7
		**Melanoma**	4.1 (18%)	6.0 (16%)	2.1
		**Colorectal**	2.6 (12%)	3.8 (10%)	2.1

*Complete prevalence rates and rates of prevalence in those at 10 years or more postdiagnosis are corrected prevalence estimates. Cancer site data are for complete prevalence.

**Relates to the proportion of complete cancer prevalence accounted for by each subset of cancer prevalence (limited duration or cancer site).

**Figure 2:**
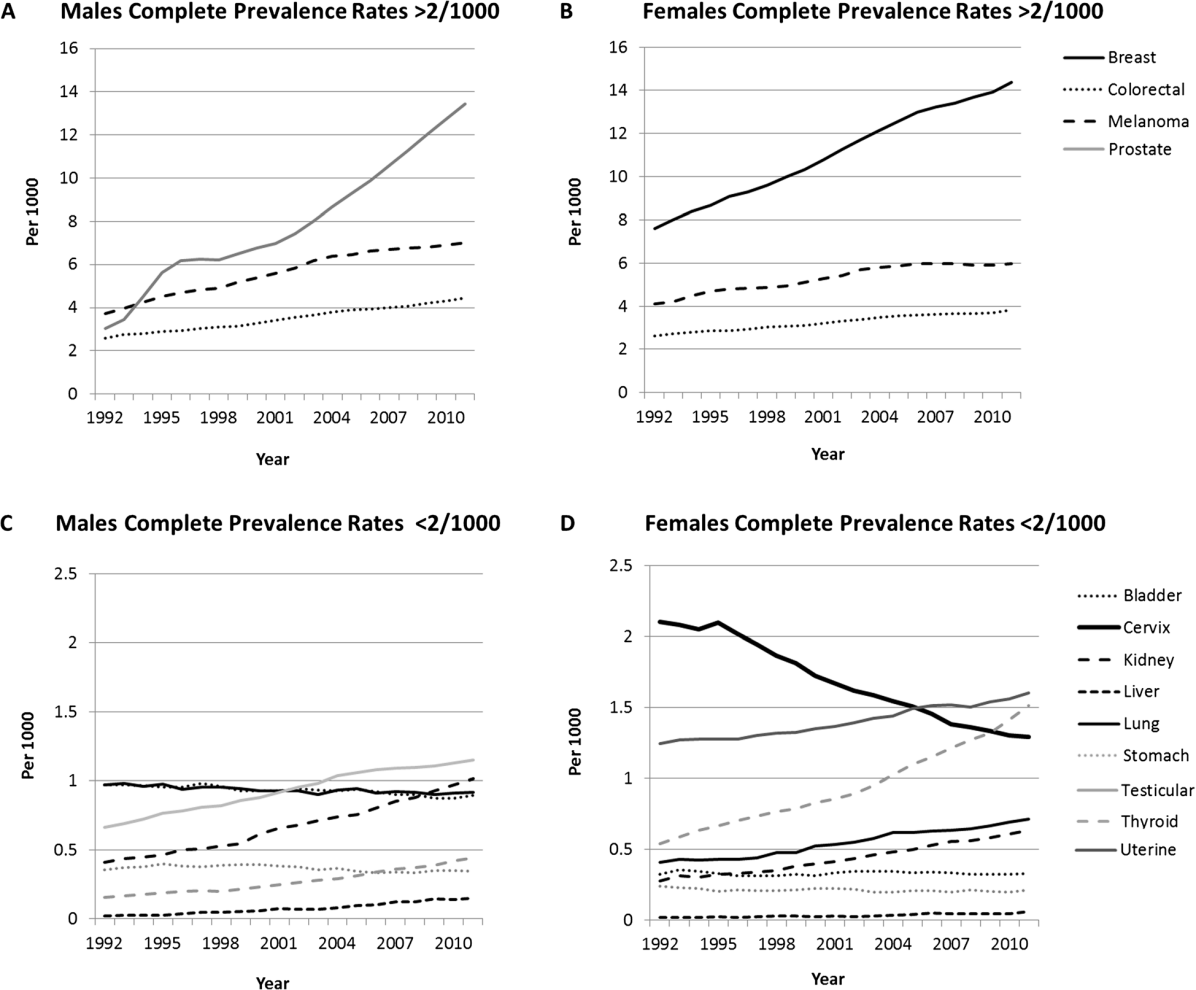
**Complete prevalence rates in males and females for selected cancers, 1992–2011.** These figures are based on complete corrected prevalence estimates.

**Figure 3:**
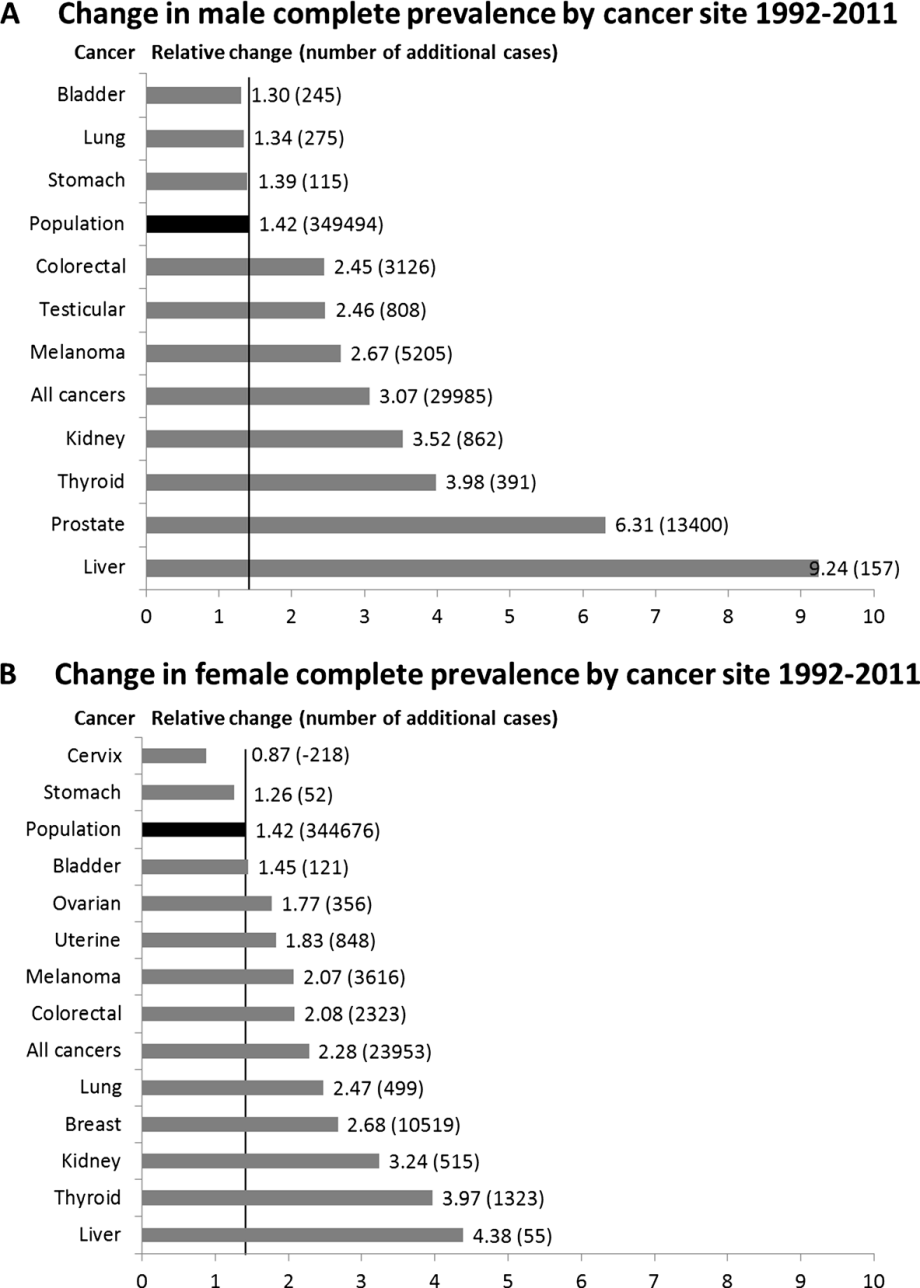
**Change in complete cancer prevalence for males and females by cancer site, 1992–2011.** These figures are based on complete corrected prevalence estimates. The bars indicate the relative change in the complete prevalence of each cancer between 1992 and 2011. Population growth is shown by the black bar. Where the relative change in the cancer is less than the relative change of the population (indicated by the black line), the prevalence rate has decreased. The numbers in brackets indicate the absolute number of additional cases since 1992.

For the majority of cancers, the complete prevalence rate increased linearly in both males and females; however, rates of cancer of the cervix, stomach, and bladder decreased (Figure 2). The prevalence of prostate cancer increased rapidly in the early 1990s, plateaued, and then steadily increased (Figure 2). The complete prevalence rate of lung cancer showed a small decrease in males (0.62 to 0.56 per 1,000) and a steady increase in females (0.26 to 0.45 per 1,000). Similar trends were seen for other periods since diagnosis (data not shown). Of the less-prevalent cancers (<2 per 1,000) the most dramatic relative changes in prevalence for males and females between 1992 and 2011 were for cancers of the liver, thyroid, and kidney (Figure 3). Forty-five and 44% of the increase in complete cancer prevalence in males and females between 1992 and 2011 can be attributed to prostate and breast cancer, respectively (Figure 3).

### Cancer-related hospitalizations

**Figure 4:**
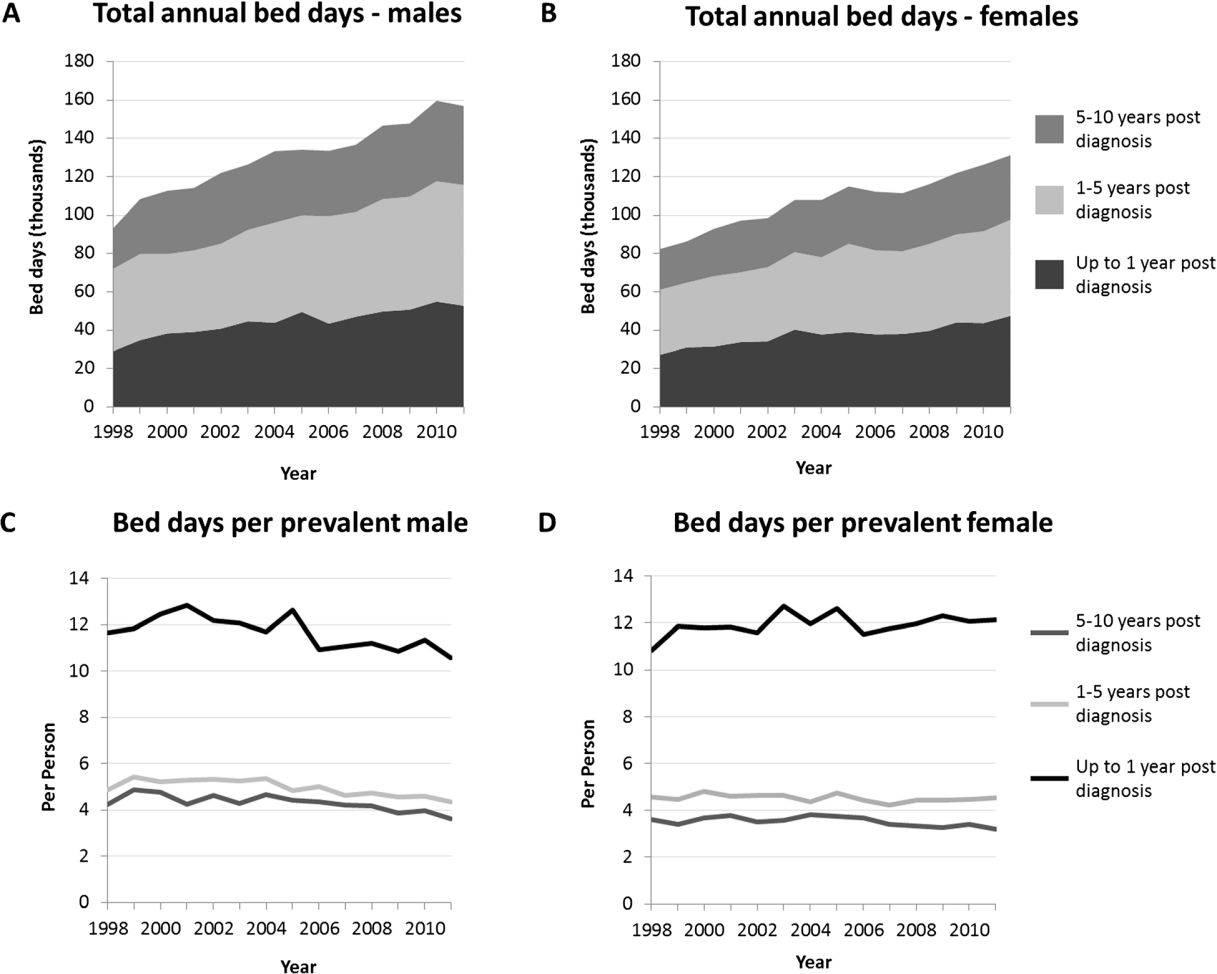
**Bed days per individual (C/D) and total annual bed days (A/B) in males and females in 2011 by time since diagnosis.**

**Figure 5:**
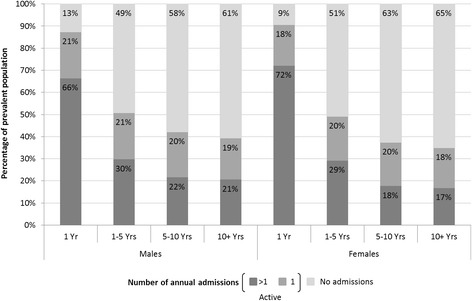
**Proportion of prevalent individuals with a cancer-related hospitalization in 2011 by time since diagnosis.**

The number of total bed days per prevalent case remained relatively constant between 1998 and 2011, with a slight downward trend in males; however, over the study period, the absolute number of bed days increased 81 and 74% in males and females diagnosed within one year, respectively (Figure 4). The population increased by 30% over this time. The number of bed days per prevalent individual varied by time since diagnosis, with males and females within a year of diagnosis having an average of 10.5 and 12.1 bed days, respectively, in 2011. For those diagnosed between one and five years and between five and 10 years earlier, annual bed days dropped significantly, with 4.4 male and 4.5 female bed days and 3.6 male and 3.2 female bed days, respectively. The proportion of prevalent cases with a cancer-related hospital admission in 2011 also decreased with duration of prevalence (Figure 5), as did the proportion with more than one hospital admission within a year. Forty-seven and 42% of the 2011 prevalent males and females, respectively, who had ever been diagnosed with cancer (uncorrected prevalence) had at least one-cancer related hospital admission in 2011, with 27 and 23% having more than one admission (data not shown).

## Discussion

Since the early 1990s the number of people living in Western Australia with a history of cancer has increased by a magnitude of 2.5-fold, easily outpacing population growth. The majority of these individuals have been living with cancer – or beyond cancer– for more than five years, and were diagnosed with prostate, breast, melanoma, or colorectal cancer, which cumulatively account for two-thirds of all cancers. Cancer prevalence in WA and the mix of types of cancer are similar to those reported in a global study, with one in every 60 to 70 people living in the United States, Australia, and Europe having had a cancer diagnosis with the previous five years and breast and prostate carcinomas being the most prevalent cancers. The relative prevalence of melanoma stands out in WA, with a prevalence that surpasses that of colorectal cancer, the second-most prevalent cancer globally and third-most prevalent cancer in men and women in WA in 2011 [16,17].

### Understanding the increase in prevalence

While the drivers for the increase in prevalence are both incidence and survival, a 2013 comparative analysis of cancer statistics in the United States, Italy, Nordic countries, Australia, and France found variation in prevalence rates between countries to be more strongly associated with incidence [16,17]. We found the majority of the increase in cancer prevalence in males and females to be due to prostate and breast cancers, respectively, much of which may be explained by increasing incidence of these cancers [1] in an aging population but also improved survival following earlier detection and advances in medical treatment. The “hump” seen in the prevalence of prostate cancer in the 1990s (Figure 2A) may be explained by the increased diagnosis of prostate cancer after the prostate-specific antigen (PSA) test became available in the late 1980s [18] and actively promoted in the early-to-mid 1990s [19]. In the longer term, assuming screening is not leading to overdiagnosis – “the diagnosis of a cancer that would otherwise not go on to cause symptoms or death” [20] – screening should have little impact on incidence. The PSA test however, has been shown to overdiagnose prostate cancer, with one study reporting overdiagnosis as high as 84% [18,20]. Notably, a 2002 comparative study of prostate cancer prevalence rates in Europe concluded that much of the variation in prevalence was likely to be due to differential uptake of PSA testing [8]. Screening mammography for breast cancer, introduced nationally in Australia in the early 1990s, has also been shown to overdiagnose cancer, particularly ductal carcinoma in situ [21,22]. A 2014 paper quantified the extent to which overdiagnosis may occur, reporting that among 40-, 50-, and 60-year-old women undergoing annual mammography over 10 years, up to 11, 3–14, and 6–20 per 1,000 women, respectively, would be overdiagnosed and needlessly treated [23]. The magnitude to which overdiagnosis, also reported in other cancers including thyroid cancer and melanoma [20], has increased cancer prevalence is unknown, but raises concerns of unnecessary medical treatment – and associated harms– in an increasingly burdened health system.

The large relative increase in the prevalence of thyroid cancer, consistent with a marked increase in incidence globally over the past 40 years, is thought to be due to the incidental discovery of nodules during ultrasound for unrelated conditions, as well as changes in diagnostic thresholds [24]. Incidence also explains the rise in the prevalence of liver cancer, although survival has also improved [14]. Lung cancer, accounting for around 9% of all incident cancer cases, is a comparatively less-prevalent cancer due to its high mortality rate, representing 19% of all cancer deaths in 2009 [1]. The prevalence of lung cancer increased in women, while in men it showed a slight downwards trend, reflecting changes in the incidence of lung cancer, mirroring smoking trends in men and women 30 to 40 years ago [25]. Decreasing trends in both cervical and bladder cancer reflect falling incidence; however, the change in cervical cancer has occurred as a result of a successful screening program to detect and treat precancerous changes, while the fall in bladder cancer incidence is largely attributed to changes in coding, complicating the interpretation of trends [14].

### Health service needs of prevalent individuals

The rising prevalence of cancer translates into a growing burden on hospitals, with a 70 to 80% increase in the total number of cancer-related hospital bed days over 13 years, more than twice the relative population growth. As expected the demand on health services is highest in those accessing acute cancer care services within one year of diagnosis; however, we found that more than a third of prevalent individuals were still accessing cancer-related hospital services for their primary cancer more than 10 years after their diagnosis, with 40 to 50% of all prevalent cases having at least one cancer-related hospitalization within a year. While not all cancer services are delivered in hospital, this measure of hospital service use could be considered a surrogate measure of “active” prevalence, that is, cancer cases requiring ongoing treatment. In 2002, Brameld et al. estimated that 25% of prevalent individuals in 1998 were “active;” this is significantly lower than our estimate, however the Brameld et al. calculation was based on the proportion of patients that die from their cancer, a method the authors accepted would underestimate the need for ongoing health care [4]. Furthermore, Brameld used more complex analytic methods to estimate complete prevalence due to the shorter look-back period and therefore results may not be comparable [4]. These high rates of active prevalence (30 to 40% of complete prevalence) even in individuals more than 10 years after diagnosis reinforce cancer as a chronic disease.

The changing health needs of prevalent cases go beyond hospital care to monitoring and surveillance and the management of long-term and late effects from cancer care. Many people living beyond cancer face physical and psychological long-term and late effects of their cancer; the most commonly reported include fatigue, sexual dysfunction, decreased participation in activities, and musculoskeletal problems [7]. The type and extent of effects vary by the type of cancer, treatment, and age. Previous reports also cite poorer management of comorbidities, including diabetes and cardiovascular disease in cancer survivors compared to the nonprevalent population. Primary care services will be increasingly relied upon to provide holistic management for the growing body of cancer survivors [26].

### Limitations

The extent to which cancer hospitalization data reflect hospital cancer service use depends on coding and administrative practices. In WA, but not in all Australian jurisdictions, the majority of chemotherapy patients are classified as admitted patients, while the majority of radiotherapy patients are nonadmitted (outpatients) as they are throughout Australia [27,28]. As such, our cancer-related hospitalization data will include most chemotherapy but not radiotherapy services. This is a significant limitation, with around 35% of all cancer patients accessing radiation services at some time during their illness [29]. In addition, our measure of active prevalence does not reflect the proportion of prevalent cases requiring ongoing cancer services outside the hospital system. Many individuals are likely to continue to access pharmacological, adjuvant, palliative, and primary care services as a result of their cancer in the absence of a hospital admission.

The precision of our results also depends on the integrity of the cancer and hospital morbidity data, as well as the migration of individuals out of the state. Under-reporting of cancer cases has the potential to underestimate prevalence, while loss to follow-up due to migration may overestimate survival and therefore prevalence. Additionally, our study was limited to individuals resident in WA at the time of their cancer diagnosis, with individuals who migrated into the state included in population statistics but unable to contribute to cancer prevalence, potentially underestimating prevalence rates and cancer-related bed days. Furthermore, the estimated complete prevalence did not account for differential survival, assuming survival occurred as per 2001 rates, effectively overestimating prevalence rates in the early years.

Our corrected estimates for complete prevalence assumed a 29-year look-back to be sufficient. This is consistent with Capocaccia and De Angelis, who found a 30-year look-back period provided acceptable prevalence estimates in populations where the average age of prevalent cases is quite high [9]. However, a small underascertainment bias may remain for cancers with high survival and relatively earlier age of occurrence (e.g., breast and cervical cancer and cancers diagnosed in childhood) [9]. While the technique to adjust prevalence is simplistic, it is appropriate for data where full capture is available but would not be suitable for data where the longest look-back period does not infer full capture. In such cases a more complex algorithm would be required based on relative survival in the population concerned.

## Conclusion

Our data paint a bleak picture of steadily increasing prevalence and cancer burden on hospital services, beyond population growth; a demand that is in part due to success in improving cancer survival and general life expectancy in the community. To stall trends in prevalence rates in an aging population, continued efforts are required to prevent cancer through modifiable risk factors, as has been achieved to some extent with lung cancer in males. Furthermore, rising prevalence will continue to demand better use of health resources. Steps should be taken to address and understand the extent of cancer over diagnosis, particularly of prostate cancer, and its impact on prevalence and cancer service use. In the longer term, developments in genomics may allow for better targeted screening and more accurate predictions of tumor behavior that mitigate overdiagnosis and prevent overtreatment [20,30]. Additionally, our data highlight the need for the consideration of cancer services beyond acute care, meeting the needs of the thousands of individuals facing long-term adverse outcomes from cancer treatment, particularly for those most prevalent cancers.
